# Treatment of a Large Gluteal Hydatid Cyst in Syria Using a Manual Negative Pressure Wound Therapy Device (PragmaVAC)

**DOI:** 10.1055/s-0044-1788773

**Published:** 2024-09-25

**Authors:** M. Netfagi, H. Alshaer, A. Abbara, M. Hariri

**Affiliations:** 1 Department of Surgery, Rahma Darkoush Hospital, Darkoush, Syria; 2 Pragmatic Innovation Inc, Mississauga-Toronto, Ontario, Canada; 3 Imperial College, London, United Kingdom; 4 Department of Education, Syrian Board of Medical Specialties, Gaziantep, Turkey

**Keywords:** hydatid disease, Syria, innovation, gluteal abscess, VAC pump

## Abstract

The lungs and liver are the most common sites of hydatid cysts, but they may also be found in other organs. We report the case of a lady in her 50s who presented to a hospital in northwest Syria with a large swelling in the right gluteal region. This was diagnosed as a gluteal abscess, and an incision and drainage were performed. Intraoperatively, a hydatid cyst germinal membrane was noted. She had the required imaging to exclude other cysts, which revealed a liver hydatid cyst of 7.5 cm, which was treated with PAIR (puncture, aspiration, injection, and re-aspiration) and albendazole. The residual wound was closed using a manual vacuum-assisted closure (VAC) pump for seven days. A key lesson is that a hydatid cyst in the gluteal muscles, though rare, should be considered in the differential diagnosis. We also show that the innovative use of a manual VAC pump can be used to support closure of large hydatid cyst cavities in muscles.

## Introduction

Hydatid disease is an endemic parasitic infection caused by the canine tapeworm, mostly *Echinococcus granulosus* , and it is an important public health problem in the Mediterranean, Middle East, Africa, South America, Asia, and Australia. 1 The definitive hosts for *E. granulosus* are dogs and the intermediate hosts are most commonly sheep though other animals including cattle, horses, goats, and camels are also potential intermediate hosts. 1 Humans become infected when they ingest eggs; the larva then burrow through the intestinal wall, entering the bloodstream. They may enter the portal venous system affecting the liver (in 60–70% of cases) or the pulmonary circulation affecting the lungs (5–27% of cases) manifesting as cysts. 1 Hydatid cysts can, however, occur in other organs including the central nervous system, bone, kidney, or in the subcutaneous tissue. 2 3 Subcutaneous hydatid cysts are rare but present the same concern about the risk of rupture resulting in anaphylaxis or the presence of daughter cysts. 1 Treatment approaches include conservative, drug treatment, surgery, or PAIR (puncture, aspiration, injection, re-aspiration) depending on the stage. Here we present a case from a hospital in northwest Syria noting the successful use of a manual vacuum-assisted closure (VAC) pump for wound management.

## Case Report

A woman in her 50s with a body mass index (BMI) of 35 kg/m ^2^ presented to a hospital in northwest Syria with a 10-day history of a painful right gluteal mass. She had no medical history, and there was no history of a recent intramuscular injection. She lived with her extended family and did not smoke or drink. She took no regular medication and had no known drug allergies. On physical examination, there was a swelling in the upper lateral quadrant of the right gluteal region with signs of erythema, tenderness, and warmth. Abdominal, chest, and neurologic examinations were normal.

**Fig. 1:**
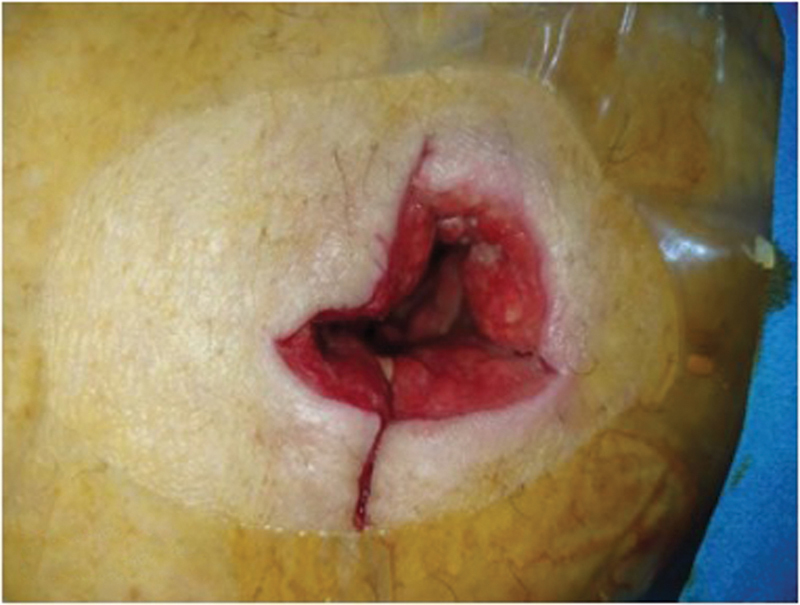
Gluteal hydatid disease cavity postsurgery.

**Fig. 2:**
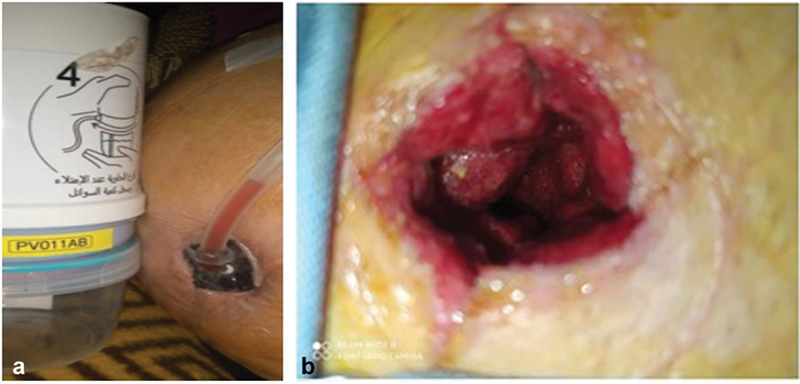
( **a** ) PragmaVAC device and pump. ( **b** ) Wound after PragmaVAC device removal.

**Fig. 3:**
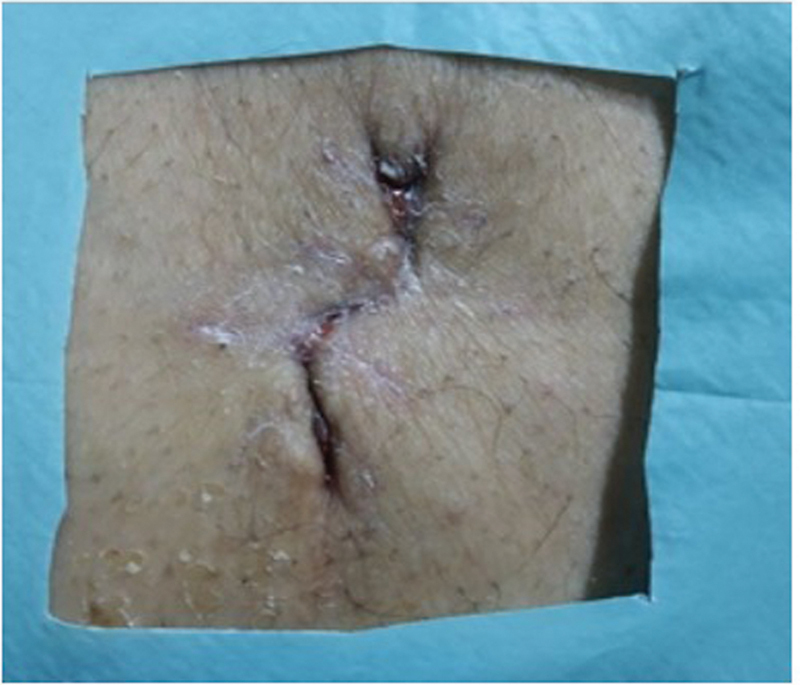
Final outcome of the wound.

She was initially diagnosed with a pyogenic gluteal abscess, and an incision and drainage were done on the same day. During the operation, about 500 mL of fluid was drained from the abscess and a hydatid cyst germinal membrane was noted during irrigation and suction of the cavity, raising suspicion of hydatid disease ( Fig. 1 ). Postoperatively, the cavity was treated with daily dressings for 4 days, then manual negative pressure wound therapy (NPWT) was applied using a PragmaVAC device for 7 days due to the size of the wound ( Fig. 2a ). Afterward, an endoscope was used to show the entire cavity, which revealed good and healthy granulation tissue without infection ( Fig. 2b ). The wound was closed by stitching on layers ( Fig. 3 ).

**Fig. 4:**
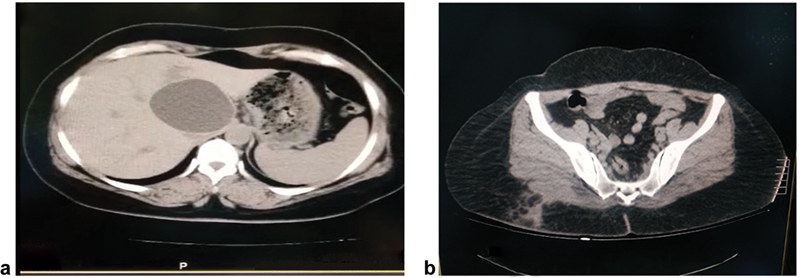
( **a** ) Computed tomography (CT) scan showing the hepatic hydatid cyst. ( **b** ) CT scan showing gluteal abscess postsurgery.

Due to the diagnosis of a likely hydatid subcutaneous cyst, the patient had a chest X-ray, which was unremarkable, and then went on to have an ultrasound and then computed tomography (CT) scan of her liver; this revealed the presence of an active solitary hydatid cyst without daughter cysts in the liver of 7.5 cm ( Fig. 4a ). This was treated with PAIR with adjunctive albendazole 400 mg twice daily for 3 months as per the WHO standard guidance for stage CE3a for a cyst greater than 5 cm. 4 5 An ultrasound of the gluteal area was performed after 3 weeks, and it showed no residual cavity and no other cysts. After about 1 month, a gluteal CT scan was performed for the area to ensure cavity closure and to look for other cysts ( Fig. 4b ). The patient was reviewed 1 month later and was noted to have no complications or evidence of recurrence.

## Discussion

Subcutaneous hydatid cysts in the gluteal region are an extremely rare event even in areas where the disease is endemic. 2 It usually presents as a chronic, painful lump, and hydatid disease must be considered in the differential diagnosis in endemic areas. 2 Serology may also be helpful although it may not be widely available. Imaging can be helpful as it may show the characteristic features of a hydatid cyst, which include double echogenic lines of the cyst wall separated by a hypoechogenic layer, multiple-echogenic foci, detachment of the endocyst from the pericyst, and the presence of daughter cysts. 6

For a large hydatid cyst in the gluteal region that is symptomatic or at risk of rupture, most experts would recommend complete excision. Interestingly, Kayaalp et al's review of 22 cases of subcutaneous hydatid cyst noted that none were associated with anaphylaxis, something that is of concern in liver or lung hydatid cysts. In this case, identifying it to be a hydatid cyst avoided the unnecessary use of antibiotics and meant that adjunctive medical treatment with antihelminthic drugs, such as mebendazole or albendazole, is used to reduce risk of local recurrence. 1 This is usually continued for at least 3 months with patients followed up at intervals to monitor progress or complications.

In this case, we also highlight the therapeutic potential of NPWT to support closure of the cavity. Both randomized controlled trials and retrospective cohort studies report benefits such as reduced wound volume, accelerated granulation formation, and a lower incidence of postoperative complications including infection, dehiscence, and necrosis. 7 8 Its clinical applications currently encompass exposed bone or joints, deep sternal wounds, open abdomen, or intra-abdominal infections, as well as ulcers from pressure injuries, vascular insufficiency, or as complications of diabetes. 7 Manual NPWT (PragmaVAC) is a novel device that generates negative pressure by manual pressing, without electrical power. The generated pressure level is –100 mm Hg on average, which is compatible with the standard commonly used in treatment of open wounds. It consists of a medical grade standard plastic bellow (pump) attached to the wound dressing via a tube. 8 The NPWT technique proved to be effective in such cases, especially where the cavity is large or the patient has a large BMI. It is also useful in low-resource settings where electricity sources may be unreliable.

## Conclusion

Despite being rare, primary subcutaneous hydatid cyst should be considered for the differential diagnosis of soft-tissue masses or abscesses particularly for patients who live in regions where hydatid disease is endemic. Manual NPWT (PragmaVAC) can be effective in treating large cavities without any adverse consequences, help accelerate granulation tissue formation, and significantly reduce in wound dressing frequency. Manual NPWT can be a practical and cost-effective device in settings of conflict where electricity is in short supply, making it a useful adjunct in such settings.
